# A Few Practical Points in Infantile Anemia

**Published:** 1904-12

**Authors:** 


### A FEW PRACTICAL POINTS IN INFANTILE ANEMIA.

Infantile anemia always manifests distinctive, objective and subjective symptoms of malnutrition, of which the most prominent is a lowered blood standard. Disease of childhood shows more pronounced changes in the blood than does disease of the same class in the adult; therefore a careful study of the blood is immediately necessary and yields points of greatest importance in deciding the best method of treatment.

There is still a great deal to be determined about the marks of distinction between the normal and abnormal conditions of the blood in children; much has been developed, but there is still much to be learned. In the transition from infancy to childhood, the process of the development of the blood is going on and it is often hard to determine whether a certain case is normal or abnormal in the condition of the blood. For instance, the percentage of hemoglobin which is, in proportion, higher at birth than in adult life, may fall within the first few weeks of life to fifty per cent., and still not be abnormal. It is, therefore, for the busy practitioner, no less than for the one not so proficient in hematology to have some simple, reliable and easily practical methods of ascertaining the exact blood condition. When this knowledge is obtained a diagnosis can be made. To obtain this practical knowledge, no special skill is required; the ordinary use of the microscope, hemoglobinometer, and harmocytometer, can with a little practice obtain the necessary data. It is now conceded that aside from chlorosis nearly all cases of anemia in children are of secondary origin, consequently it is not difficult to ascertain the causes. The tendency may be transmitted from the anemic, poorly nourished patient; in sufficient quantity an improper kind of food is usually the chief cause. The infectious or constitutional conditions, such as rickets, syphilis, tuberculosis, malaria, rheumatism, etc., are responsible for the great majority of cases of secondary anemia, and usually present one or more distinctive symptoms indicating their origin. The thing most desired in the treatment of these conditions is, naturally, to remove the cause, which is sometimes possible, but not always; a careful study of the blood should be the first step, after which its proper treatment, for by this means one can often remove the subjective symptoms of the anemia, thereby making the patient more comfortable as well as reinforcing the treatment of the cause.

In the endeavors to restore the normal standard of the blood in cases of secondary anemia, dietetic and hygienic measures are of the greatest importance. A careful study of many cases shows conclusively that a large proportion of the cases which owe their origin to conditions prevalent among the poorer classes—improper food, poor air, lack of exercise—are the prime causes. A correction of these defects with proper feeding, fresh air and tonic, will bring about the desired results without the necessity of much drug medication. But in those cases in which the anemia is secondary to the infections, the dietetic or hygienic measures can be supplemented by the application of proper medication; the treatment then becomes one of removing the cause of the anemia, at the same time reinforcing the system with proper nutrition. The most frequently employed drugs as blood reconstructors, are iron and arsenic, but their field of usefulness is limited. They undoubtedly produce a tonic effect by stimulation, but lack the proper elements to build up the newly born cells as the result of this stimulation, consequently their therapeutic value is limited and something more complex is required. Too short a space is allowed to enumerate the many cures laid down by various cliniations in the treatment of anemia. All have virtue more or less, none are complete. Most of the tonics of iron will increase the blood-cells without a corresponding increase in the hemoglobin, consequently, many of the new-born cells never reach maturity but become shriveled, disintegrated or paralyzed as a result of the malnutrition. With this clinical picture before me I naturally sought for something that fully covered the field, in other words, a tonic, stimulant and complete food. The combination of the three making the essential whole I found in Bovinine, and its employment in many cases has proven it to be a most valuable dietetic and therapeutic agent.—T. J. BIGGS, M.D.

